# Context-dependent roles of YAP/TAZ in stem cell fates and cancer

**DOI:** 10.1007/s00018-021-03781-2

**Published:** 2021-02-13

**Authors:** Lucy LeBlanc, Nereida Ramirez, Jonghwan Kim

**Affiliations:** 1 Department of Molecular Biosciences, The University of Texas at Austin, Austin, TX 78712 USA; 2 Interdisciplinary Life Sciences Graduate Program, The University of Texas at Austin, Austin, TX 78712 USA; 3 Harvard Medical School, 25 Shattuck St, Boston, MA 02115 USA; 4 Center for Systems and Synthetic Biology, The University of Texas at Austin, Austin, TX 78712 USA

**Keywords:** Hippo signaling, YAP, Yap1, TAZ, Development, Stem cells, Cancer, Apoptosis

## Abstract

Hippo effectors YAP and TAZ control cell fate and survival through various mechanisms, including transcriptional regulation of key genes. However, much of this research has been marked by conflicting results, as well as controversy over whether YAP and TAZ are redundant. A substantial portion of the discordance stems from their contradictory roles in stem cell self-renewal vs. differentiation and cancer cell survival vs. apoptosis. In this review, we present an overview of the multiple context-dependent functions of YAP and TAZ in regulating cell fate decisions in stem cells and organoids, as well as their mechanisms of controlling programmed cell death pathways in cancer.

## Introduction

YAP and TAZ are effectors of the Hippo pathway, functioning as transcriptional co-regulators that play roles in various cellular contexts. YAP was initially recognized for its roles in inducing hepatomegaly and apoptosis resistance in mice and for its ortholog Yorkie (Yki) positively regulating organ growth in *Drosophila* [1]. Since YAP and TAZ lack DNA-binding domains, they partner with transcription factors (TFs) (e.g., TEAD1-4, AP-1) to modulate the expression of their target genes [2, 3]. YAP and/or TAZ in complex with TEAD typically associate with distal enhancers and occasionally with promoters, and their functions include controlling transcriptional pause release, H3K27 acetylation, and nucleosome occupancy to influence target gene expression [4–6]. This complex modulates genes involved in cell fate specification, including lineage markers, self-renewal factors, and apoptosis-related genes, which results in the maintenance or loss of cell identity depending on the physiological context.

**Fig. 1:**
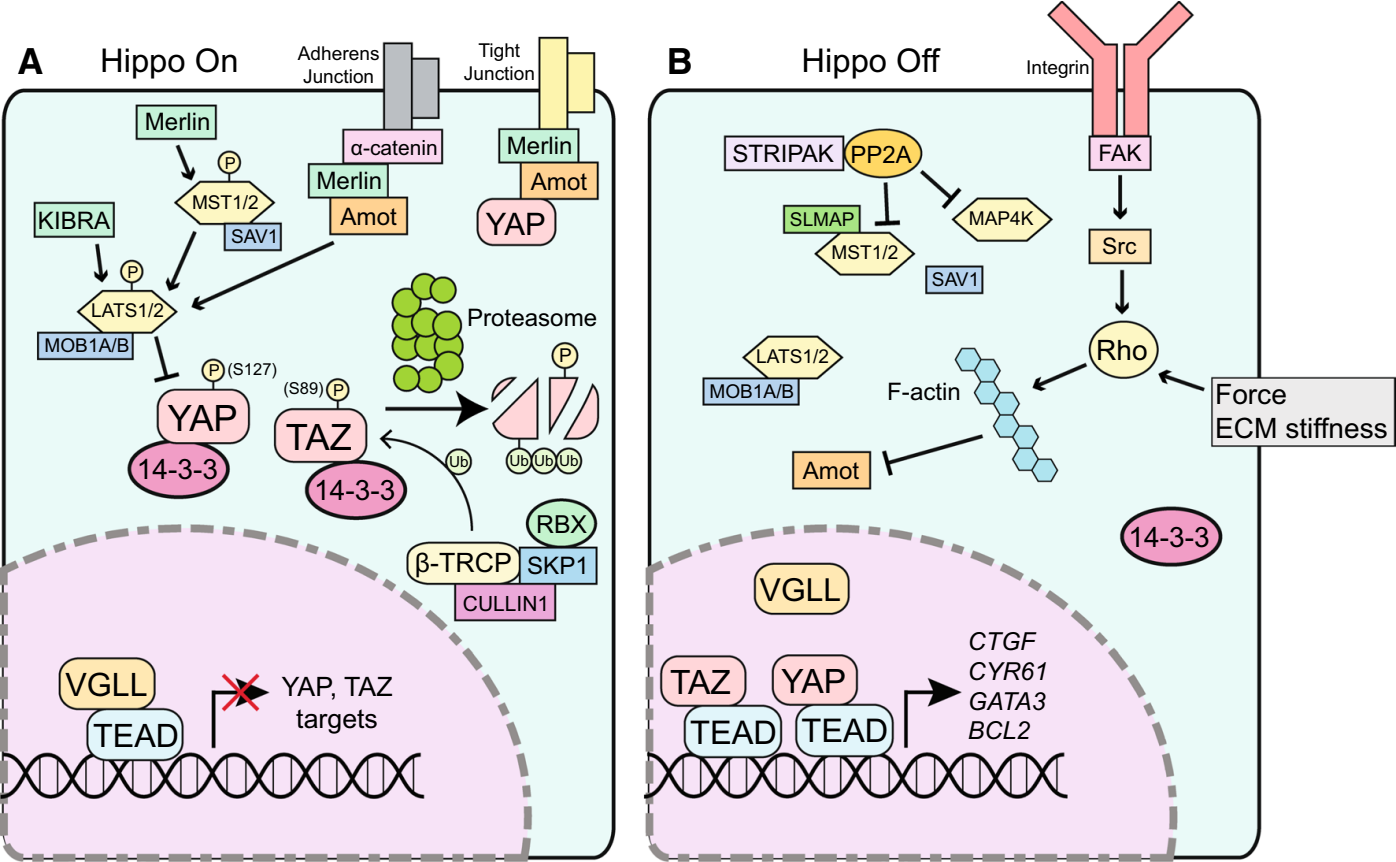
A graphical summary of Hippo signaling. **a** When Hippo signaling is on, MST1/2 kinases phosphorylate LATS1/2 kinases, activating them to phosphorylate YAP and TAZ at multiple sites. These phosphorylation events are sufficient for sequestration in the cytoplasm via 14-3-3. Additional phosphorylation events lead to polyubiquitination and proteasome-mediated degradation. In the nucleus, VGLL competes for binding to TEAD, further reducing YAP and TAZ activity. **b** When Hippo is off, frequently by activation of Rho kinase or inactivation of Hippo kinases, YAP and TAZ translocate to the nucleus to regulate target genes in concert with TEAD factors and other TFs

YAP and TAZ are primarily controlled by the Hippo pathway, which is composed of a series of kinases that activate one another and negatively regulate YAP and TAZ’s protein stability and nuclear localization [7]. These kinases were initially identified as tumor suppressors in *Drosophila* that negatively regulated the transcriptional activity of Yki, which is equivalent to mammalian YAP, and the TF scalloped, equivalent to TEAD [8, 9]. These kinases are in turn influenced by various upstream signals including G protein-coupled receptors (GPCRs), Wnt proteins, cell–cell contact, and mechanical force [10]. GPCR ligands activate YAP and TAZ by inhibiting upstream kinases such as LATS1/2 and stimulating actin polymerization, which tends to promote YAP activity [11–13]. Since its discovery, Hippo signaling has been shown to regulate a myriad of cellular processes, including embryogenesis, tumorigenesis, chromatin remodeling, metabolism, and the inflammatory response [8, 10, 14, 15]. We have presented a brief summary of Hippo signaling in Fig. 1.

YAP and TAZ are notable in their ability to change both cell fate and survival. Depending on the context, they can either maintain a stem or progenitor state or stimulate differentiation and morphogenesis. We consider cell fate and survival to be linked, as morphological and biochemical similarities between apoptosis and differentiation have caused some to call them “twins separated at birth” [16]. Numerous pathways influence cell survival, but we will focus on the intrinsic apoptotic pathway, driven by activation of caspase-9 [17–19]. The rate of caspase-9 activation is impacted by the relative abundance of Bcl-2 family proteins, which influence mitochondrial homeostasis; although many exist, anti-apoptotic BCL-2 and pro-apoptotic BAX are often transcriptionally regulated by YAP [20]. Though many studies cite that YAP regulates apoptosis, there have been few if any systematic analyses comparing the mechanisms in different contexts.

Contextual dissection of YAP and TAZ (both as individual proteins and comparing them as paralogs) is key, because prior literature has shown many conflicting roles for these proteins in cell fate changes, both in self-renewal vs. differentiation and survival vs. cell death [21], whereas other processes like cell motility and regulation by the cytoskeleton seem to show more consensus. It also remains controversial whether they are functionally redundant or fulfill unique roles [22]. Stem cells and cancer are relevant systems for this undertaking, as they share capacity for differentiation and self-renewal as well as similar signaling pathways [23–25]. Thus, this review will expound on how YAP and TAZ regulate cell fate in stem cells and apoptosis in cancer, and what confounding variables have contributed to contradictions within the literature.

## Functions of YAP in embryogenesis, stem cell fate and survival, and organoids

### In vivo roles of YAP in the developing mouse and human embryo

#### Phenotypes of Yap1 insufficiency in the mouse embryo

The controversy surrounding Yap1 in development typically involves its roles in early embryogenesis. *Yap1*^−/−^ embryos undergo developmental arrest around E8.5; nevertheless, the precise mechanisms underlying the development defects remain unclear [26]. Despite typically undergoing successful gastrulation, these embryos show a consistently short and wide body axis, caudal dysgenesis, lack of chorioallantoic fusion, and yolk sac vasculature defects. On the other hand, *Wwtr1*^−/−^ (encoding Taz) embryos are viable, although they develop multicystic kidney disease as early as E15.5, and the majority of them do not survive to adulthood [27–29]. This stark difference in phenotype strongly implies non-redundancy between YAP and TAZ in development. During the earliest stages of embryogenesis, loss of either gene individually does not cause a pre-implantation defect; however, dual knockout (KO) *Yap1*^*−/−*^;*Wwtr1*^−/−^ embryos die before the 32 cell stage, implying that they are essential but redundant for morula formation [30]. While more sophisticated conditional KOs have shown that Yap1 plays critical roles in the cranial neural crest, female reproductive tract, and heart valves, these phenotypes tended to be late or post-natal, and it would be fruitful to apply conditional KO in the early embryo or placenta to revisit the null phenotypes [31–33].

#### Roles of Yap1 in mouse embryogenesis

One possible mechanism for these null phenotypes may be cell–cell competition regulated by Yap1 in the pre-implantation mouse embryo [34]. Beginning at E3.75, Yap1 starts to translocate from the cytoplasm to the nucleus of some cells in the inner cell mass (ICM), resulting in variable Tead activity that controls expression of Myc and core pluripotency factors. By E4.0, ICM cells that still have primarily cytoplasmic Yap1 undergo apoptosis, and this process ensures a high-quality epiblast. Therefore, it is conceivable that embryos entirely lacking Yap1 undergo developmental errors due to lack of quality control, leading to body axis disruption and other phenotypes, as quality control is particularly crucial at this early stage of development [35]. However, that does not explain the high penetrance of these phenotypes, as a defect in quality control should not affect all embryos.

Given the recent revelation that many embryonic phenotypes can in fact be traced to placental abnormalities [36] and the fact that the placenta originates from the trophectoderm (TE) of the blastocyst, it is worth considering whether Yap1 or Taz null phenotypes originate from TE lineage defects. Indeed, the Yap1–Tead4 complex has been shown to be critical for TE specification. In the outer cells of the blastocyst, simultaneous lack of Hippo signaling [37] and the presence of Notch signaling [38] allows for nuclear localization of Yap1. This in turn allows the Yap1–Tead4 complex to activate expression of Cdx2 and Gata3, specifying the TE. Consequently, it was initially thought that Tead4 is absolutely required for TE formation. However, Kaneko & DePamphilis rebutted this view by showing that Tead4 is dispensable for TE specification when two-cell and eight-cell embryos are cultured in conditions that relieve oxidative stress [39]. Despite strong evidence that Yap1 and Tead4 are key for TE development under physiological conditions, it still remains unknown whether the embryonic phenotypes of null embryos are caused by defects in extraembryonic tissues or are merely concurrent with them.

#### YAP in the human vs. the mouse embryo and placenta

Like in the mouse, YAP is essential for the development of the human TE and placenta, and its expression is negatively correlated with pregnancy disorders like preeclampsia [40, 41]. The YAP–TEAD4 transcriptional program was recently shown to maintain human trophoblast stemness in primary cytotrophoblasts and repress cell fusion [40]. This was confirmed in another study using both a mouse model and human trophoblast stem cells (TSCs) derived from patients who had experienced pregnancy loss [42]. However, the precise roles of YAP in the human ICM are contested. Yap1 is still inactive and cytoplasmically sequestered in the ICM of the mouse blastocyst by E3.5, whereas it is nuclear in the TE. This is partially established by the Par-aPKC system, which polarizes the blastocyst and thus suppresses Hippo signaling in the outer cells but activates it in the ICM [43]. In contrast to mice, in the human blastocyst, YAP undergoes nuclear accumulation in both the ICM and TE 5–6 days post-fertilization [44]. Whether this may be due to a mismatch in the staging of the human and mouse blastocyst remains undetermined. Nevertheless, YAP has also been shown to sustain pluripotency in human embryonic stem cells (hESCs), and YAP overexpression promotes acquisition of naïve pluripotency, which does not occur in mouse embryonic stem cells (mESCs) [44, 45]. If it were merely an embryo staging issue, YAP would not promote naïve pluripotency in one species but drive differentiation in another. These concerns about the contrasting roles of YAP in mouse vs. human have prompted investigation using in vitro methods that model the blastocyst, namely ESCs and induced pluripotent stem cells (iPSCs).

### In vitro roles of YAP in stem cell self-renewal and differentiation

#### Controversies about mouse Yap1 in self-renewal vs. differentiation

In mESCs, it was initially argued that Yap1 is essential in the nucleus for maintaining self-renewal downstream of LIF by sustaining the expression of core pluripotency factors [46, 47]. This was unusual, because in the mouse ICM, Yap1 is known to be cytoplasmic as mentioned earlier. Further studies using multiple CRISPR KOs have shown that mESCs lacking Yap1 are functionally indistinguishable from WT in regards to proliferation and maintenance of stemness [45]. Furthermore, like in the mouse ICM, Yap1 was found to be cytoplasmic and only translocated to the nucleus upon differentiation. Overexpression of Yap1 also leads to flattened colonies and premature upregulation of lineage markers [45]. These findings are more consistent with in vivo observations, because during post-gastrulation, around E7.5, Yap1 is critical for expression of the early endoderm regulon [48]. Finally, reducing nuclear YAP by inhibiting RHOA GTPase in mouse E3.5 blastocysts leads to the downregulation of TE markers and upregulation of ICM markers, showing that Yap1 may actually transcriptionally antagonize ESC core factors in mouse [49].

However, there continues to be a debate, as KO of upstream Hippo kinases leads to a differentiation defect during teratoma formation that is rescued when Yap1 is knocked down, implying that Yap1 represses lineage specification in teratomas [50]. The contradictory nature of these reports may be due in part to the use of different mESC lines, methods, or failure to test non-cell-autonomous effects of Yap1. Surprisingly, it has been recently shown that during reprogramming, cells that overexpress Yap1 promote the reprogramming of nearby somatic cells that are not overexpressing Yap1; indeed, cell-autonomous expression of Yap1 actually inhibits the acquisition of pluripotency [51]. Additionally, constitutive Yap1 expression promotes astrogenesis in neighboring cells during late embryonic development by upregulating Cntf and represses astrogenesis in a cell-autonomous manner [52]. Thus, whether a cell expresses Yap1 may not be as important as whether its neighbor expresses Yap1. Collectively, these studies show that Yap1 is likely unimportant for maintenance of mESC self-renewal, but it does participate in lineage specification, and can influence cell fate in a non-cell-autonomous manner.

#### YAP promotes self-renewal and naïve pluripotency in hESCs

Meanwhile, for hESCs, YAP generally inhibits differentiation and promotes self-renewal. Although this may appear to be the reverse of its roles in mESCs, one may initially hypothesize that this is likely due in part to the distinct developmental stages that mouse and hESCs represent on the pluripotency spectrum. After all, both hESCs and iPSCs in standard culture conditions represent a primed pluripotent state, which is more similar to the post-implantation E4.5–5.5 epiblast. Contrarily, in standard mESC culture, cells exhibit naïve pluripotency reminiscent of the E3.5 pre-implantation ICM [53]. The primed state is marked by X chromosome inactivation and highly inefficient germline contribution, whereas both X chromosomes are active in naïve ESCs and pre-implantation chimeras are readily formed. However, primed vs. naïve pluripotency cannot explain the differential impacts of YAP in mouse vs human, as overexpression of Yap1 in mESCs causes premature upregulation of lineage markers, whereas overexpression of YAP in hESCs causes them to enter a naïve state of pluripotency [44, 45]. We propose that it is more likely that differences in binding partners, genomic targets, sequence, isoform, or even the degree of overexpression may contribute to these opposite results rather than developmental staging alone. To our knowledge, depletion or ablation of YAP in naïve hESCs has not yet been performed; thus, it is unclear whether YAP is essential for maintaining human naïve pluripotency or merely acquiring it.

**Fig. 2:**
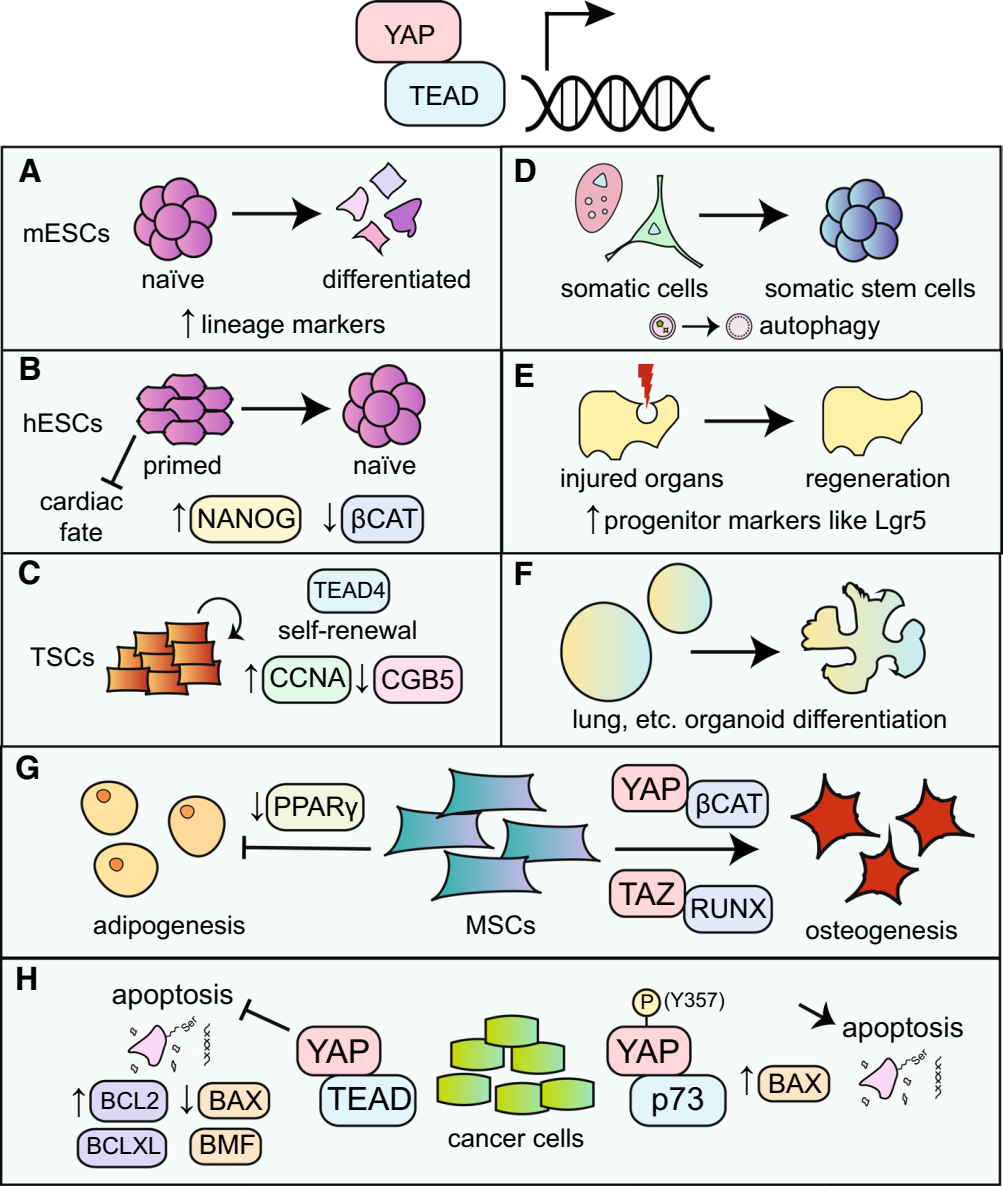
An overview of YAP-mediated cell fate changes. **a** Yap1 promotes differentiation of mESCs via upregulation of lineage markers, particularly trophoblast. **b** YAP promotes naive pluripotency of hESCs via upregulation of core factors and suppression of β-catenin activity. **c** In tandem with TEAD4, endogenous YAP promotes self-renewal of TSCs in both human and mouse by activating stemness markers. **d** Ectopic expression of YAP allows dedifferentiation of somatic cells like neurons to a more stem-like state. **e** YAP is essential for regeneration in some organs (lung, liver, retina, and intestines) by facilitating temporary cell fate switch to a progenitor-like state after organ injury. **f** In some organoids (lung, kidney, and esophageal), YAP expression promotes branching and morphogenesis. **g** MSCs are pushed to the osteogenic fate on stiff substrate when YAP is active. **h** YAP can either prevent apoptosis in cancer cells by binding to TEAD, or promote apoptosis by binding to p73

YAP promotes pluripotency in hESCs through a variety of mechanisms. It is known that SMADs sustain the expression of NANOG in the absence of WNT3, but when WNT3 is present, SMADs cooperate with β-catenin to induce mesendoderm marker expression [54]. YAP impairs the recruitment of SMADs and reduces occupancy of RNA polymerase II (RNAPII) at the *WNT3* locus, preventing expression of WNT3 [55]. YAP expression appears to be highly correlated with the self-renewing state: growing hESCs in 3D culture increases both YAP and core factor expression, and verteporfin-mediated inhibition of YAP strongly reduces expression of both core and naïve markers [56]. Intriguingly, YAP also maintains self-renewal of the human TE by binding to the promoters of *CCNA* and *CDK6* in cytotrophoblasts [40]. This would imply that in the human blastocyst, both the ICM and the TE identities are sustained by YAP, compared to only the TE in the mouse. We have summarized the effects of YAP in cell fate changes in Fig. 2, although this covers not just its effects in stem cells, but in all other major cell fate decisions covered in this review as well.

#### Early lineage specification and differentiation in both human and mouse

Further research confirms the importance of Yap1 in mediating the development of all three germ layers, particularly the neuroectoderm in mouse as well as repressing the mesendoderm lineage in human. During differentiation, Rassf1a promotes the phosphorylation of Yap1, which causes it to switch binding partners from Tead2 and β-catenin to p73 and initiate lineage-specific transcriptional programs [57]. However, not all three germ layers are equally favored, and it seems that in mice, Yap1 favors the ectoderm lineage [58]. Mechanistically, activation of Yap1 (for example, via KO of upstream Hippo kinases Mst1/2) leads to the formation of novel phase-separated Med1-positive super-enhancers that promote the expression of ectoderm markers [55] while downregulating mesendoderm markers [58]. In humans, YAP appears to inhibit the cardiac fate as culturing *YAP*^*-/-*^ hESCs in Activin A results in the spontaneous derivation of beating cardiomyocytes. Therefore, YAP’s complexity and its associated signaling crosstalk seem to depend on species.

Yap1 seems to regulate apoptosis in ESCs, as well. Apoptosis occurs naturally during differentiation in part to cull mESCs that fail to exit self-renewal efficiently, and *Bax*^*−/−*^*Bak*^*−/−*^ double KO cells, which cannot undergo apoptosis, display marked defects in embryoid body and teratoma formation [59]. Apoptosis during differentiation occurs not only during LIF withdrawal but also during other modes of differentiation, such as epiblast [60]. Differentiating mESCs that lack Yap1, regardless of their ultimate fate, undergo 2–3-fold higher rates of apoptosis due to defective expression of Bcl-2 and Mcl1 [61]. *Yap1*^−/−^ mitochondria are more sensitive to apoptotic stress, leading to hyperactivation of caspase-9, and this can be rescued by overexpression of Bcl-2 or Bcl-xL. Intriguingly, double knockdown of YAP and TAZ in hESCs results in cell death that can also be rescued by BCL-xL overexpression, implying that they redundantly guard against apoptosis during primed pluripotency [62]. Although it has not yet been tested whether YAP protects against apoptosis during hESC differentiation, YAP appears to fill a dual role in stem cell survival and identity that varies depending on species and lineage.

### YAP and somatic stem cells

#### Reacquisition of a stem-like state via YAP overexpression

Though not as potent as ESCs, many lineage-restricted progenitor/stem cells exist in post-natal tissues, and their cell fate is often guided by YAP. It has recently been appreciated that activation of YAP helps mediate the reversion of differentiated cells to a more primitive, stem-like state. Organ regeneration after injury is mediated by the expansion and differentiation of tissue-specific somatic (sometimes referred to as adult) stem cells. Unfortunately, in humans, somatic stem cells are often scarce, limiting regeneration. Typical approaches for stem cell therapy involve reprogramming of patient-derived fibroblasts into iPSCs; however, iPSCs can form tumors when introduced into adult tissues if not completely differentiated [63]. Thus, the derivation and transplantation of non-tumorigenic somatic stem cells have been proposed as an alternative to iPSCs.

Fascinatingly, brief overexpression of YAP or TAZ for 5–15 days can convert luminal mammary cells, neurons, and pancreatic acinar cells into their respective lineage-restricted somatic stem cells [64, 65]. For example, it is possible to reconstitute a cleared mammary gland with a YAP-induced mammary stem cell-derived organoid, and the resultant regenerated gland can differentiate into clusters of milk-secreting alveoli in a pregnant mouse [65]. In the adult mouse heart, constitutively activating YAP partially reprograms cardiomyocytes into a fetal-like, proliferative state via chromatin remodeling [66]. Furthermore, overexpression of YAP rejuvenates senescent mesenchymal stem cells and alleviates symptoms of osteoarthritis in mice, and conditional KO of YAP in astrocytes inhibits regeneration of axons after spinal cord injury [67–70]. These findings open tantalizing possibilities for safer stem cell therapy.

#### Endogenous YAP and regeneration

Aside from intentional and ectopic overexpression of YAP to generate somatic stem cells, reverse senescence, or promote regeneration, it has become increasingly clear that normal organ repair after injury involves transient YAP activation. For example, colonic regeneration in a mouse model of colitis and restoration of the adult heart post-myocardial infarction involves YAP and Lgr5^+^ stem cells in intestinal tissues, and organoids undergo expansion after injury in a YAP-dependent manner [71–75]. Also, YAP is critical for regeneration in the lung after damage to the alveolar epithelium, in the liver for hepatocytes to reprogram into biliary progenitors after the injury, and in the retina for Müller glia to proliferate after eye injury [76–81]. In several of the aforementioned examples, the mechanism by which YAP promotes regeneration is not elucidated. However, at least in pancreatic and mammary contexts, dedifferentiation caused by YAP or TAZ activation appears to be dependent on increased autophagy [82].

This has been observed in other species as well. In the mouse liver, acute inactivation of YAP efficiently dedifferentiates hepatocytes into self-renewing liver progenitor-like cells [83]. Also, caudal fin regeneration in zebrafish occurs through Yap1 promoting osteoblast differentiation, which is reminiscent of how Yap1 and Taz promote osteoblast activity in mice [84, 85]. These studies strongly evince that YAP serves as a common mechanism for differentiated cells to revert to more primitive states in response to tissue damage and participate in regeneration, and this capacity of YAP presents an invaluable opportunity for patient-specific stem cell therapy without needing to generate iPSCs.

### YAP functions in organoid cultures

#### Contextual roles of YAP in promoting morphogenesis in organoids

Organoids have been proposed as an in vitro method to explore how pluripotent or somatic stem cells form tissues and organs in 3D [86]. They circumvent some of the ethical and financial concerns of in vivo models while also allowing for straightforward genetic manipulation and patient-specific genotypes [87]. Intriguingly, multiple organoid models have shown diverse roles of YAP in either promoting or blocking differentiation and morphogenesis. For example, an organoid model of severe liver damage reveals that the activity of YAP is essential for TET1, a DNA demethylase implicated in reprogramming, to epigenetically reset ductal cells, allowing them to differentiate into cholangiocytes and hepatocytes [88]. In lung organoids, nuclear YAP is found at invasive tubular structures, and knockdown of YAP disrupts the formation of these structures [89]. Similarly, in kidney organoids, nuclear YAP is observed at the tips of elongating ureteric buds, which further supports its roles in cell migration and tissue morphogenesis [90]. Conversely, inhibition of the retinoic acid pathway prevents alveolar epithelial differentiation by augmenting YAP nuclear translocation [91], whereas YAP promotes epithelial differentiation in mouse esophageal organoids [92]. Additionally, retinoic acid inhibition promotes a regenerative state in intestinal organoids and blocks differentiation by maintaining active nuclear YAP [93]. Thus, organoid models also suggest context-dependent roles of YAP in driving or suppressing differentiation.

Organoids have also allowed insights into mechanical and paracrine regulation of YAP. In intestinal organoids, high matrix stiffness increases YAP activity and promotes intestinal stem cell expansion [94]. When mouse intestinal organoids are co-cultured with fibroblasts, the fibroblasts secrete prostaglandin E2, promoting the expansion of the stem cell population via Yap1 [95]. Furthermore, bile acids promote intestinal organoid growth, self-renewal, and fate specification, likely through YAP [96]. Similarly, matrix stiffness promotes liver organoid formation via focal adhesion kinase and YAP [97]. In brain organoids cultured in hyaluronic acid, a common component of the extracellular matrix (ECM), YAP is indispensable for forebrain patterning [98]. Given that embryogenesis is also known to be influenced by the extracellular matrix and paracrine signaling [99], it would be intriguing to investigate how both the matrix and paracrine factors influence YAP functions in vivo and in vitro.

#### Using organoids to study roles of YAP in early embryogenesis

As ethical concerns make it difficult to molecularly dissect the roles and regulation of YAP in the human embryo, researchers have started using 2D micropatterning and 3D models such as blastoids, peri-implantation organoids, and gastruloids to understand germ layer specification, pre-gastrulation development, and body plan specification [100]. Despite the existence of these models, they have not been used to elucidate embryogenesis-related functions of YAP in greater depth. Recently, hESCs have been used to generate a trophoblastic spheroid that has been proposed as a blastocyst surrogate; YAP expression is more intense and nuclear in these spheroids, and inhibition of YAP leads to defects in attachment and outgrowth [101]. This is promising, but follow-up studies are needed. Gastruloids in particular represent an exciting avenue for elucidating the regulation of YAP during embryogenesis in mouse vs. human. Placental organoids should also be pursued due to the essential roles of YAP and TEAD4 in TE specification in both species [42].

## Functions of TAZ and canonical downstream targets in cell fate

### Biochemical differences between YAP and TAZ

#### Differences in protein stability

Many researchers in the field of Hippo signaling consider YAP and TAZ to have very similar functions, even considering them redundant to the point where they are referred to interchangeably as YAP/TAZ [64, 102]. As paralogs with high (~ 50%) sequence identity, these two proteins possess both redundant (i.e., they can genetically compensate for one another) and non-redundant roles [2]. They have a similar target gene profile, and they are redundant in the oviduct, heart, urinary tract, and other organs [31, 103–105]. However, they have numerous context-specific differences and fulfill distinct roles during embryogenesis and cell fate specification.

One of the starkest differences between YAP and TAZ is the presence of an additional N-terminal phosphodegron in TAZ, leading to a much shorter half-life [106]. In mouse embryonic fibroblasts, the half-life of TAZ is a mere 2 h, whereas YAP protein levels remain stable for 6 h [107]. YAP and TAZ are frequently regulated by altering their protein stability, although they are sometimes impacted differently. For example, nonreceptor tyrosine kinase PYK2 enhances tyrosine phosphorylation of TAZ but not YAP in triple-negative breast cancer, increasing its stability, and their expression is positively correlated in primary breast tumors [108]. Furthermore, in both mouse and human, YAP promotes the degradation of TAZ via GSK3 and HSP90, whereas TAZ does not affect the protein abundance of YAP [109]. This is controversial as in human corneal fibroblasts, TAZ knockdown leads to elevated YAP levels but not vice versa [110]. Having both paralogs—one relatively stable, the other rapidly fluctuating, and both possibly engaging in negative feedback—may be advantageous for mammalian cell homeostasis.

#### TEAD complex formation and dimerization

Aside from these differences in half-life, YAP and TAZ display different binding modes to TEAD as well as distinct, isoform-specific capacities for dimerization and phase separation. Whereas human and mouse YAP binds to TEAD in a 1:1 heterodimeric complex, the crystal structure of the mouse Taz–Tead4 is a heterotetrameric complex where two molecules of Taz bind to two molecules of Tead4, likely due to the absence of a PXXΦP motif in the Taz linker sequence [111, 112]. Furthermore, unlike YAP, TAZ overexpression can induce phase separation, allowing it to compartmentalize partner TFs and promote transcription, and TAZ can homodimerize via its coiled-coil domain, whereas YAP cannot [113, 114]. Thus, organisms may require both YAP and TAZ due to their regulation being so distinct; YAP levels may remain stable over relatively long periods, whereas TAZ levels can rapidly increase, homodimerize, and phase separate, decreasing quickly when it is no longer needed. However, their comparative abilities to dimerize and phase separate remain controversial. A long isoform of YAP known as YAP2L can homodimerize both in vitro and in vivo, while shorter isoforms cannot [115]. Also, YAP does have an intrinsically disordered region, and upon hyperosmotic stress, YAP forms liquid-like condensates around super-enhancers [116]. Future research focusing on isoform-specific functions of YAP and TAZ should be carried out to explain these inconsistencies and determine in what physiological contexts they are most relevant.

#### Domain architecture, binding partners, and unique targets

**Fig. 3:**
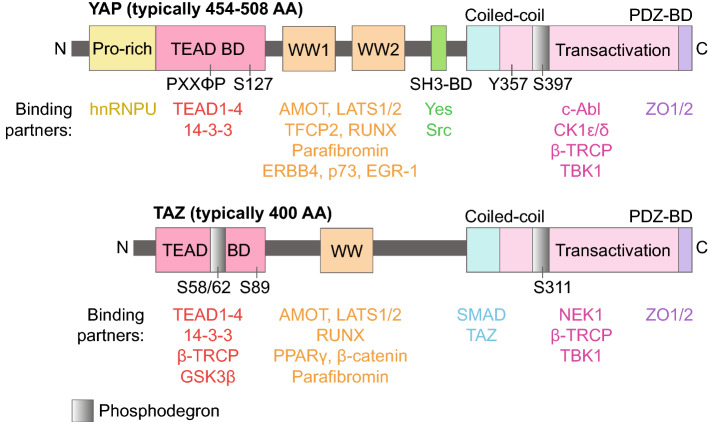
YAP and TAZ domain architecture. Overview of YAP and TAZ domains and phosphodegrons as well as key binding partners. YAP’s PXXΦP motif as well as regulatory serines and a tyrosine that are known to be phosphorylated are also indicated. *BD* binding domain

These biochemical differences between YAP and TAZ as well as differences in primary sequence contribute to differences in binding partners and transcriptional targets. Aside from an additional phosphodegron in TAZ, these two proteins have slightly different domain architecture. In YAP, from *N*- to *C*-terminus, this includes a proline-rich domain, a TEAD-binding domain, 1–2 WW domains that interact with PPXY motifs on proteins like LATS, p73, and many others, an SH3-binding domain, a coiled-coil domain, a transactivation domain, and a PDZ-binding domain [117–123]. TAZ only has one WW domain and lacks a proline-rich domain or SH3-binding domain. We have detailed the domain structure of YAP and TAZ and indicated several of their associated binding partners in Fig. 3.

As a result of differences in sequence and domain architecture, TAZ, but not YAP, binds to Smad4 in mesenchymal stem cells, facilitating its nuclear localization, and enhancing osteogenesis in partnership with RUNX2 [124]. TAZ also promotes osteogenesis in bone marrow mesenchymal stem cells and periodontal ligament cells [125, 126]. YAP also increases osteogenesis, yet it works with a different set of binding partners, and in tandem, they regulate osteoblast and osteoclast activity [85, 127]. TAZ blocks adipogenesis by acting as a co-repressor of PPARγ via direct interaction and repression of its targets [128]. Intriguingly, parafibromin, a nuclear scaffold, interacts with YAP after parafibromin is tyrosine phosphorylated, but after dephosphorylation, it interacts with TAZ instead [129]. Finally, these distinct binding partners result in some unique transcriptional targets. The integrin ITGAV is a target of TAZ, but not YAP, in hepatocellular carcinoma, and it is associated with poor clinical outcome, indicating unique roles of TAZ in oncogenesis [130]. In ovarian cancer, ANGPTL4 is a direct target of TAZ but not YAP [131]. Intriguingly, in non-small cell lung cancer, their overall transcriptional programs have little overlap: YAP activates genes involved in cell cycle progression whereas TAZ activates genes critical for migration, resulting in differential sensitivity to chemotherapeutics [132]. Therefore, their unique transcriptional targets can have biologically significant consequences. Future research should combine structural and in vivo approaches to elucidate the functions of more of their targets and, most urgently, how TAZ and YAP can regulate different sets of genes despite their similarities.

### TAZ in cell fate specification

#### In development and stem cells

As noted earlier, YAP KO is embryonic lethal after E8.5 due to gross body axis defects, yolk sac vascular defects, and failure to undergo chorioallantoic fusion, whereas TAZ KO leads to kidney and lung defects as well as post-natal lethality. They also have unique roles in maintaining primed pluripotency. Cytoplasmic TAZ promotes the self-renewal of both hESCs and mouse epiblast stem cells (mEpiSCs) via sequestration of β-catenin, whereas nuclear translocation of TAZ results in mEpiSC differentiation [133]. However, in that study, YAP did not associate with β-catenin in either hESCs or mEpiSCs. Thus, the two are not functionally redundant in early embryogenesis or in primed pluripotency, though it is unknown whether TAZ also participates in the primed-to-naïve transition as YAP does.

#### In somatic cells

Despite dramatic disparities in embryonic phenotype which have not yet been explored in mechanistic detail, most of the research comparing YAP and TAZ in differentiation has focused on adult tissues. TAZ is much less studied than YAP, and they are rarely directly compared, limiting our ability to assess their redundancy. However, it is known that YAP and TAZ exert complementary time-sensitive roles in the lungs, whereas in fibroblasts, T cells, and myoblasts, YAP and TAZ play somewhat opposing roles. Volckaert et al. in 2019 found that cytoplasmic Yap1 suppresses Fgf10 by promoting the degradation of β-catenin during early lung development, enabling alveolar epithelial differentiation, whereas Taz promotes the differentiation of alveolar type 1 cells [134]. Furthermore, TAZ, but not YAP, is essential for the differentiation of alveolar type 2 to alveolar type 1 cells, and conditional deletion of TAZ reduces lung regeneration and increases fibrosis [135].

Meanwhile, in corneal fibroblasts, their roles are contrasting rather than complementary. Knockdown of YAP in these cells leads to reduced CTGF and Smad2/3/4 expression, whereas knockdown of TAZ upregulates CTGF and αSMA, and simultaneous depletion causes cell death much like in hESCs [110]. TAZ is required for pro-inflammatory TH17 helper T-cell differentiation and prevents differentiation toward immunomodulatory Treg cells [136]. Meanwhile, YAP inhibits naïve T-cell differentiation regardless of ultimate fate, and the deletion of YAP enhances differentiation to TH1, TH17, TH2, and Treg fates under polarizing conditions [137]. Whereas both Taz and Yap1 promote myoblast proliferation, only Taz binds to Tead4 in myoblasts to enhance myogenic differentiation [138]. In sum, despite observations that YAP and TAZ are similar enough to be redundant in many circumstances, their biochemical and regulatory differences are sufficient to result in distinct consequences for cell fate specification, as summarized in Table 1. Future studies should address the physiological implications and structural underpinnings of their non-redundancy.

**Table 1:** Biochemical, genetic, and cell fate-related differences between YAP and TAZ

Context/aspect	TAZ	YAP	Citation
Domain architecture	TEAD-binding, WW domain, coiled-coil, transactivation domain, PDZ binding (400 AAs)	Pro-rich, TEAD-binding, 1–2 WW domains, SH3-binding, coiled-coil, transactivation domain, PDZ binding (488 AAs)	[193]
Half-life	2 h in C3H/10T1/2 cells; < 1 h in U2OS cells	> 6 h in C3H/10T1/2 cells; ~ 1.5 h in U2OS cells	[107, 194]
Binding mode with TEAD	2:2 heterotetramer	1:1 heterodimer	[111, 195]
Self-dimerization	Yes	No (Murakami et al.), Yes for YAP2L isoform (Khanal et al.)	[114, 115]
Phase separation upon overexpression	Yes	No (Lu et al.), Yes (Cai et al.)	[113, 116]
Mutual regulation	TAZ does not affect YAP expression or stability (Finch-Edmondson et al.), knockdown of TAZ upregulates YAP (Muppala et al.)	YAP promotes TAZ degradation via GSK3 and HSP90	[109, 110]
Mouse embryonic null phenotype	Viable, but kidney disease present by E15.5 as well as lung defects, and post-natal survival is poor	Embryonic lethal between E9.5 and E10.5 with a shortened body axis, yolk sac vascular defect, caudal dysgenesis, and more	[26–29]
Mouse EpiSC and human ESC self-renewal	Sustains primed pluripotency by sequestering and stabilizing β-catenin in the cytoplasm; in hESCs, partners with OCT4 to repress mesendoderm genes	In hESCs, partners with OCT4 to repress mesendoderm genes; when overexpressed, promotes transition to naïve pluripotency	[44, 133, 196]
Osteogenesis in mesenchymal stem cells and periodontal ligament cells	Promotes osteogenesis and represses adipogenesis via binding with RUNX2, IRS-1, and/or Cbfα1	Promotes osteogenesis and represses adipogenesis by stabilizing nuclear β-catenin	[85, 124–127]
Early lung development	Promotes differentiation of alveolar type 1 cells, essential for lung regeneration via cell fate conversion of alveolar type 2 to type 1 cells	Suppresses Fgf10, enabling alveolar epithelial differentiation	[134, 135]
Corneal fibroblasts	Represses CTGF and αSMA, limiting YAP-mediated transdifferentiation via Smads	Activates CTGF and Smad2/3/4	[110]
T-cell differentiation	Prevents differentiation of T cells toward Treg cells and encourages TH17 helper T-cell differentiation	Inhibits differentiation of CD4 + T cells to TH1, TH17, TH2, and Treg fates and reduce tumor infiltration	[136, 137]
Myoblasts and mouse skeletal muscle tissues	Promotes myoblast proliferation, muscle growth, and myogenic differentiation, but not regeneration	Promotes myoblast proliferation and muscle regeneration, but not differentiation	[135]
Promoting apoptosis in cancer	Represses MYC and its targets in multiple myeloma	Binds to p73 and upregulates *BAX* in various cancer tissues	[142, 164, 165, 185, 197, 198]
Ferroptosis in cancer	Pro-ferroptotic via regulation of EMP1 in renal cell carcinoma or ANGPTL4 in ovarian cancer	Pro-ferroptotic via ACSL4 in colon cancer	[131, 186, 187]

## YAP/TAZ and apoptosis in cancer

### YAP as an oncogene or a tumor suppressor via apoptosis regulation

#### Cancer types where YAP reduces apoptosis

Due to metabolic and signaling similarities between cancer and stem cells, it is unsurprising that YAP regulates similar processes in the two cell types [24, 139]. YAP orchestrates many processes during oncogenesis, including metastasis, chemoresistance, and cancer stemness. Focusing on apoptosis, in most cancer types, ranging from gastric to colon to renal, YAP inhibits apoptosis, as seen in mESCs, to help drive tumor progression. Furthermore, YAP expression is generally associated with poor prognosis and heightened tumor aggressiveness in cancers like oral squamous cell carcinoma and cholangiocarcinoma [140, 141]. However, much like the disagreement about whether YAP promotes self-renewal vs. differentiation in ESCs, there have been reports where YAP has functioned as a tumor suppressor and promoted apoptosis rather than preventing it [142].

In gastric cancer, YAP reduces apoptosis by promoting mitophagy downstream of SIRT1 activity, MFN2 expression, and upregulation of growth factors, whereas the targeting of YAP increases apoptosis [143–145]. In clear cell renal cell carcinoma [146] and rhabdomyosarcoma [147], YAP is upregulated, and YAP depletion increases apoptosis and decreases proliferation, even in murine xenografts in vivo. Inhibition of YAP via the chemotherapeutic norcantharidin enhances apoptosis in non-small cell lung carcinoma [148]. Similarly, targeting YAP in liver cancer [149, 150] and breast cancer [151, 152] induces apoptosis, reduces proliferation, and increases chemosensitivity. Thus, it is evident that in many different cancer types, YAP impedes apoptosis, and its depletion is sufficient to cause apoptosis in cancer, which establishes YAP as a valuable therapeutic target in the clinic.

#### Mechanisms of apoptosis regulation via YAP

Like during mESC differentiation, YAP primarily regulates apoptosis in cancer via transcriptional regulation of anti- and pro-apoptotic genes, most commonly BCL-2 and BAX. In colon adenocarcinoma, overexpression of YAP or TAZ reduces apoptosis via the upregulation of BCL-2 and downregulation of BAX; reducing YAP levels is sufficient to trigger apoptosis [153, 154]. BAX is repressed by YAP in prostate cancer [155–157], and YAP, TEAD, and SLUG collaborate to directly repress pro-apoptotic BMF in dormant non-small cell lung cancer [158]. In senescent tumor cells, YAP sustains expression of the anti-apoptotic factor survivin [159]. YAP activates expression of BCL-xL in bladder cancer, and depletion of YAP increases apoptosis [160]. Using a hybrid spheroid model of cell competition, Liu et al. showed that glioblastoma cells express YAP heterogeneously, and YAP-low cells experience more apoptosis, reminiscent of the E4.5 mouse epiblast, though it is not specified what downstream targets are involved [161]. Thus, many of the mechanisms by which YAP influences ESC survival are shared in cancer.

#### YAP as a tumor suppressor

Although YAP has often been described as an oncogene, it can act as pro-apoptotic in select contexts. YAP’s capacity for triggering apoptosis by binding p73 and upregulating BAX has been known for nearly 2 decades [142, 162–164]. YAP switches binding partners from TEAD factors to p73 after phosphorylation by c-Abl on Y357 in response to DNA damage [165]. More recent research has shown that YAP can increase apoptosis in breast cancer [166, 167], lung cancer [168], and liver cancer [169] cells, typically downstream of or alongside apoptotic stimuli like chemotherapy. This phenomenon has even been shown in non-cancer contexts, such as damaged mouse livers where Yap1^high^ hepatocytes undergo apoptosis [170]. Intriguingly, in Huntington’s disease, YAP in conjunction with TEAD can induce endoplasmic reticulum swelling and necrosis in primary neurons [171]. It still remains unknown whether targets of the YAP-p73 complex other than BAX are relevant to promoting apoptosis as well as whether other TFs can switch YAP to a pro-apoptotic transcriptional program.

### Regulation of YAP in cancer

#### Regulation of YAP protein levels in cancer

YAP itself is positively regulated by various genes that tend to be highly expressed in cancer. Many of these oncogenes reduce apoptosis in cancer cells by increasing YAP protein levels or its stability. Overexpression of E3 ubiquitin ligase FBXW7 increases apoptosis in hepatocellular carcinoma by targeting YAP for ubiquitination and degradation, and the expression of these two proteins is inversely correlated in tumors [172]. Ankyrin repeat-containing protein Kank1, when overexpressed, reduces the proliferation and increases the apoptosis of oral squamous cell carcinoma by reducing Yap1 and Taz protein expression [141]. The knockdown of ubiquitin-specific protease USP22 causes gastric cancer cell apoptosis via a reduction in YAP protein levels [173]. Therefore, altered post-translational regulation of YAP may be a common strategy for tumors to resist apoptotic stimuli.

#### Noncoding RNAs and YAP

In addition to post-translational regulation of YAP itself, various noncoding RNAs are overexpressed in cancer and suppress apoptosis by targeting Hippo kinases to indirectly raise YAP levels. miR-25, which is overexpressed in non-small cell lung cancer and suppresses apoptosis, increases YAP1 levels by targeting LATS2 [174]. miR-224-3p also represses LATS2, and its suppression leads to apoptosis [175]. The lncRNA MALAT, which is highly expressed in pancreatic cancer tissues, also targets LATS1, and knockdown of MALAT also induces apoptosis in pancreatic cancer cells [176]. Upstream of LATS kinases is KIBRA, which is targeted by miR-21 expressed in lung adenocarcinoma tissues [177]. miR-4319, a tumor suppressor that is lost in breast and non-small cell lung cancer, increases apoptosis by targeting LIN28, leading to destabilization of RFX5, a positive regulator of YAP mRNA expression [178]. In summary, tumors manipulate both post-translational regulation of YAP as well as post-transcriptional regulation of its upstream kinases via noncoding RNAs to further their own survival. Future studies should examine whether the suppression of these noncoding RNAs can facilitate cancer treatment.

### TAZ vs. YAP in cancer

#### TAZ as an oncogene

Much like YAP, TAZ is generally considered an oncogene whose expression is correlated with poor cancer prognosis [179, 180]. Depletion or inhibition of TAZ in prostate cancer [22], breast cancer [181], or glioma [93] induces apoptosis. Additionally, there are miRNAs that repress TAZ that tend to be downregulated in cancer. For example, miR-550-1 targets TAZ in acute myeloid leukemia [112] and miR-455-3p targets TAZ in pancreatic cancer [182]. Notably, unlike YAP, TAZ has a natural antisense transcript known as WWTR1-AS1; knockdown of this transcript in head–neck squamous cell carcinoma reduces TAZ expression and increases apoptosis [183]. Though YAP and TAZ are rarely compared in parallel in any given cancer study, it has been shown that TAZ, but not YAP, is upregulated in alveolar rhabdomyosarcoma, and its depletion induces apoptosis and reduces xenograft growth [184]. Importantly, their unique roles in cancer have clinical significance. YAP^high^ cancer cell lines are sensitive to the chemotherapeutic paclitaxel, but TAZ^high^ cell lines are not, and many other cancer drugs seem to affect the two groups differently [132]. Given these findings, future studies on YAP or TAZ in cancer should strive to consider both paralogs and avoid treating them as functionally redundant.

#### TAZ as a tumor suppressor

Even though TAZ does not interact with p73 like YAP does to promote apoptosis, TAZ can act as a tumor suppressor via both apoptotic and non-apoptotic mechanisms. In multiple myeloma, the *WWTR1* promoter is hypermethylated and thus silenced; re-expression of TAZ leads to apoptosis via repression of MYC and its targets [185]. TAZ was recently discovered to promote ferroptosis, an iron-dependent form of non-apoptotic programmed cell death, in ovarian cancer [131]. Intriguingly, TAZ, but not YAP, sensitizes renal cell carcinoma cell lines to ferroptosis via regulation of EMP1, whereas YAP promotes ferroptosis in colon cancer via different targets such as ACSL4 [186, 187]. However, compared to the research on their roles in tumorigenesis, much less is known about how and why these proteins promote cell death in certain situations. Thus, additional research should be performed to determine whether it is possible to take advantage of tumor suppressive YAP or TAZ in chemotherapy.

## Future perspectives

YAP and TAZ are essential for proper development, but their precise roles can differ dramatically depending on the cellular context. They can promote regeneration and reprogramming in some tissues while driving differentiation and morphogenesis in others, and their classical categorization as oncogenes is an oversimplification given their surprising ability to act as tumor suppressors in some cancer contexts. Furthermore, YAP and TAZ crosstalk extensively with other pathways, especially canonical Wnt signaling and β-catenin, but again the nature and implications of these interactions depend strongly on tissue type. In sum, YAP and TAZ display profoundly multifaceted capabilities in regulating cell fate specification and cell survival. The controversies surrounding their roles in self-renewal and apoptosis serve as a cautionary tale for extrapolating findings concerning YAP and TAZ outside of their original biological contexts.

Great strides have been made in recent years concerning the functions and regulation of YAP and TAZ, and yet much remains unknown. Most notably, YAP’s role along the pluripotency spectrum—from naïve to primed—has not been fully elucidated, despite the availability of organoid models for embryogenesis and increasingly nuanced views of how each stage of pluripotency is acquired and maintained. Additionally, it is unknown why YAP behaves as an oncogene in some contexts, but a tumor suppressor in others, and the consequences of how programmed cell death and cell fate specification interact. Furthermore, aside from differences between YAP and TAZ themselves, functional differences between the poorly characterized isoforms of YAP or TAZ may be biologically significant. Finally, it is tempting to determine whether it is possible to harness the pro-regenerative potential of YAP-mediated reprogramming without risking tumorigenesis, and whether these YAP-mediated processes proceed via distinct or shared genomic targets and binding partners. In recent years, various small molecules, peptides, and even a genetically encoded inhibitor have been shown to antagonize or promote YAP and TAZ activity, which should facilitate future research on these proteins [188–192]. However, we also speculate that increasing awareness of non-redundant roles between YAP and TAZ will motivate the development of paralog- or even isoform-specific inhibitors.

Aside from these specific inquiries, future research on YAP and TAZ should focus on incorporating single-cell and single-molecule approaches, revisiting KO phenotypes and conflicting roles in stem cells using organoid models of the early embryo, and investigating non-cell-autonomous functions of YAP. Furthermore, we contend that differences between in vitro culture systems in different labs and seeding density also influence YAP localization and activity, and such confounding factors should be taken into more careful consideration during both experimental design and execution.
